# Differential Protein Expression Profiles of Bronchoalveolar Lavage Fluid Following Lipopolysaccharide-Induced Direct and Indirect Lung Injury in Mice

**DOI:** 10.3390/ijms20143401

**Published:** 2019-07-11

**Authors:** Xinping Yue, Jessie J. Guidry

**Affiliations:** 1 Department of Physiology, Louisiana State University Health Sciences Center, New Orleans, LA 70112, USA; 2 Department of Biochemistry and The Proteomic Core Facility, Louisiana State University Health Sciences Center, New Orleans, LA 70112, USA

**Keywords:** acute lung injury, bronchoalveolar lavage fluid, proteomics, lipopolysaccharide, Cxcl15

## Abstract

The pathogenic mechanisms of acute lung injury due to direct and indirect pulmonary insults are incompletely understood. Using an unbiased, discovery and quantitative proteomic approach, we examined bronchoalveolar lavage fluid (BALF) proteome following lipopolysaccharide (LPS)-induced direct and indirect lung injury in mice. A total of 1017 proteins were both identified and quantitated in BALF from control, intratracheal (I.T., direct) and intraperitoneal (I.P., indirect) LPS-treated mice. The two LPS groups shared 13 up-regulated and 22 down-regulated proteins compared to the control group. Ingenuity pathway analysis revealed that acute-phase response signaling was activated by both I.T. and I.P. LPS; however, the magnitude of activation was much greater in the I.T. LPS group. Intriguingly, two canonical signaling pathways, liver X receptor/retinoid X receptor activation, and the production of nitric oxide and reactive oxygen species in macrophages, were activated by I.T. but suppressed by I.P. LPS. Cxcl15 (also known as lungkine) was also up-regulated by I.T. but down-regulated by I.P. LPS. In conclusion, our quantitative discovery-based proteomic approach identified commonalities, as well as significant differences in BALF protein expression profiles between LPS-induced direct and indirect lung injury, and importantly, LPS-induced indirect lung injury resulted in suppression of select components of lung innate immunity.

## 1. Introduction

Acute lung injury (ALI)/acute respiratory distress syndrome (ARDS) is an inflammatory process of the lungs that develops in response to direct (pulmonary) or indirect (extra-pulmonary) insults to the alveolar–capillary membrane, resulting in increased permeability with subsequent interstitial/alveolar edema and diffuse alveolar damage [1]. Pulmonary and extra-pulmonary ARDS are distinct syndromes with important pathophysiologic differences [2]. The causes of pulmonary ARDS include pneumonia (most common), aspiration and lung contusion, whereas the causes of extra-pulmonary ARDS include sepsis of non-pulmonary origin (most common), shock, burn injury and mass transfusion, among others [1,2]. In pulmonary ARDS, the direct insult primarily affects the alveolar epithelium with a local alveolar inflammatory response, whereas in extra-pulmonary ARDS, the indirect insult affects the vascular endothelium by inflammatory mediators through the bloodstream, exhibiting more severe endothelial injury with increased plasma markers including angiopoietin-2 and greater degradation of endothelial glycocalyx [3,4,5,6,7].

Unbiased discovery and the quantitative proteomic approach is a powerful tool for identifying novel biomarkers and regulatory signaling networks in lung diseases including ARDS [8]. The protein expression profile of the bronchioalveolar lavage fluid (BALF) directly reflects the pathological changes in the airspace milieu in ARDS. Differential BALF protein expression was observed at different time points following the onset of ARDS [9], as well as with differing disease severity (mild vs. severe) [10]. Using isobaric tags for relative and absolute quantitation technology, Bhargava and colleagues characterized BALF protein expression profiles of ARDS survivors and non-survivors at different stages of disease progression and found that non-survivors manifested decreased expression of proteins related to coagulation, iron homeostasis and immune activation, but increased expression of proteins related to glycolysis, collagen metabolism and the actin cytoskeleton [11]. In addition, this study identified several candidate biomarkers (club cell secretory protein and thioredoxin) that can be utilized to predict survival in ARDS patients [11]. By employing shotgun proteomics, Schnapp et al. identified a total of 870 proteins in BALF from three ARDS patients and further showed that insulin-like growth factor (IGF) and IGF binding protein-3 expression levels in BALF correlated with ARDS progression [12]. The above BALF proteomic studies provided new insights into the pathogenesis of ARDS and also identified novel therapeutic targets for ARDS treatment. However, these studies did not differentiate between pulmonary and extra-pulmonary ARDS, and the differences in BALF protein expression profiles between these two ARDS sub-groups are unknown.

The goal of the current study is to examine the BALF proteome following direct and indirect lung injury using the murine lipopolysaccharide (LPS) endotoxin model. LPS is the major biologically active component and primary recognition structure of gram-negative bacteria [13,14]. The murine LPS model has been widely used to study pneumonia and sepsis through pulmonary and systemic administration, respectively, and has provided important insights into the pathogenic mechanisms that play comparable roles in human patients [15,16,17]. Results from this quantitative discovery-based proteomic study revealed commonalities as well as significant differences in BALF protein expression profiles following LPS-induced direct and indirect lung injury, and importantly, this study showed that LPS-induced indirect lung injury resulted in suppression of select components of lung innate immunity, which could contribute to the so-called “immunoparalysis” in sepsis patients.

## 2. Results

### 2.1. LPS-Induced Direct and Indirect Lung Injury in Mice

**Figure 1:**
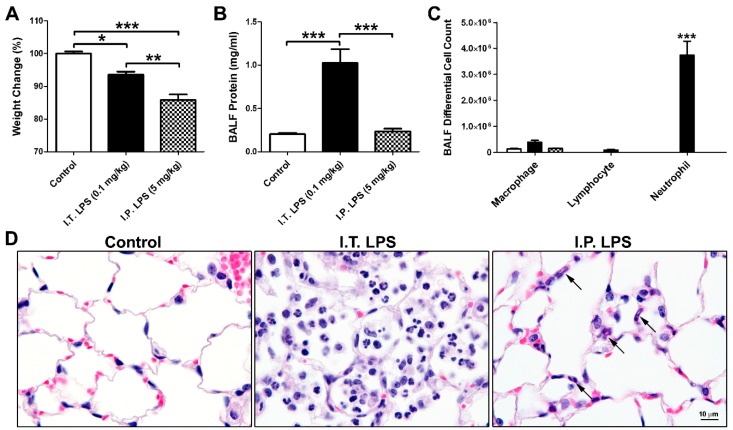
LPS-induced direct and indirect lung injury in mice. (**A**) Weight changes (% of beginning body weight) at 48 h following control, I.T. and I.P. LPS treatment in mice. N = 6 in each group; (**B**) BALF protein concentration; (**C**) BALF differential cell count. *, *p* < 0.05; **, *p* < 0.01; ***, *p* < 0.001; (**D**) H&E staining of lung sections from control, I.T. and I.P. LPS treated mice. Arrows, neutrophils.

Mice were challenged with intratracheal (I.T.) LPS at 0.1 mg/kg (*n* = 6) or intraperitoneal (I.P.) LPS at 5 mg/kg (*n* = 6) to model direct and indirect lung injury, respectively. The above dosages were chosen based on published reports [15,16], as well as our pilot studies. I.T. (*n* = 3) and I.P. (*n* = 3) administration of vehicle alone (sterile saline) were used as the control, and data from the two control groups were combined as there were no differences between the two groups. Figure 1A–C) showed weight changes (compared to beginning body weight), BALF protein concentrations, and BALF inflammatory cell profiles at 48 h following control or LPS treatment. Consistent with published reports [15,16], I.T. LPS treatment resulted in significant increases in BALF protein concentration (Figure 1B) and neutrophil cell count (Figure 1C). In contrast, although mice receiving I.P. LPS suffered greater weight loss (Figure 1A), there were no significant increases in either BALF protein concentration or neutrophil numbers compared to the vehicle treated controls (Figure 1B,C). Although BALF protein concentration was not increased in the I.P. LPS treated mice, as revealed by our proteomic analysis (below), BALF protein expression profiles of the I.P. LPS group were significantly different from those of the controls. Hematoxylin and eosin (H&E) staining of lung tissue sections (Figure 1D) confirmed the accumulation of neutrophils in the alveoli following I.T. LPS challenge. In contrast, I.P. LPS treatment did not result in neutrophil recruitment into the alveolar space; however, neutrophils can be found within the alveolar septa (arrows in Figure 1D).

### 2.2. BALF Proteomic Analysis

**Figure 2:**
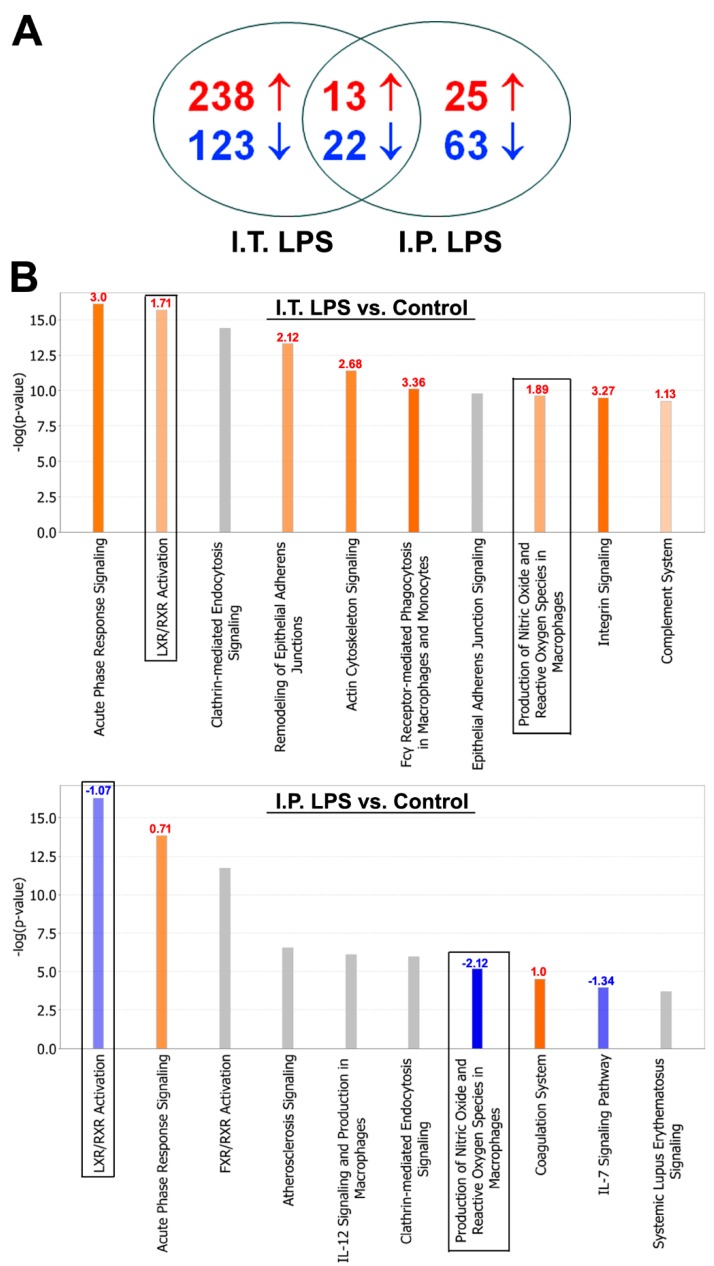
Proteomic profiling of BALF from mice treated with I.T. and I.P. LPS compared to the controls. (**A**) Number of up- and down-regulated proteins in BALF following I.T. (*n* = 4) and I.P. (*n* = 3) LPS treatment compared to the controls (*n* = 3); (**B**) Top-10 enriched canonical signaling pathways identified by Ingenuity Pathway Analysis. Orange, activated; blue, inhibited; grey, no activity pattern available. Z-score was placed on top of each bar. Positive z-score, activation; negative z-score, inhibition. Boxed pathways, differentially regulated pathways between I.T. and I.P. LPS groups compared to the controls.

We performed proteomic profiling of BALF samples from three groups of mice using the tandem mass tag (TMT) 10-plex platform: control mice (*n* = 3 with I.T. instillation of sterile saline), I.T. LPS group (0.1 mg/kg, *n* = 4), and I.P. LPS group (5 mg/kg, *n* = 3). The total identified and quantitated proteins in this assay was 1017, greater than most published reports on BALF proteome [9,10,11,12]. In addition, another 117 proteins were identified but not quantitated (Supplementary Table S1). The two LPS treatment groups shared 13 up-regulated and 22 down-regulated proteins (Figure 2A and Table 1). Among them, molecules related to bronchial and type II alveolar epithelial cell functions including cell adhesion molecule 1 (Cadm1), chloride intracellular channel protein 5 (Clic5) and surfactant protein B (Sftpb) were reduced in both treatment groups, whereas lactotransferrin (Ltf) and resistin-like alpha (Retnla), involved in lung innate immunity, were up-regulated by both I.T. and I.P. LPS challenges. There were also significant differences in BALF protein expression profiles between I.T. and I.P. LPS groups. As shown in Table 1, there were 10 proteins that were up-regulated by I.T. LPS but down-regulated by I.P. LPS, and 4 proteins that were down-regulated by I.T. LPS but up-regulated by I.P. LPS.

Figure 2B showed the top-10 enriched canonical signaling pathways revealed by Ingenuity Pathway Analysis (IPA) following I.T. and I.P. LPS challenges compared to the control group. Acute-phase response signaling was activated by both I.T. and I.P. LPS; however, the magnitude of activation is much greater in I.T. LPS group with a z-score of 3.0 compared to the I.P. LPS group with a z-score of 0.71 (Figure 2B). This finding was not surprising as I.T. LPS directly damaged the alveolar epithelium and led to an intense local inflammatory response (Figure 1).

Intriguingly, two canonical signaling pathways activated by I.T. LPS were suppressed by I.P. LPS (Figure 2B, boxed pathways). These two pathways were liver X receptor/retinoid X receptor (LXR/RXR) activation pathway involved in the regulation of lipid metabolism and the pathway involved in the production of nitric oxide (NO) and reactive oxygen species (ROS) in macrophages. The up- and down-regulated proteins in these two pathways by I.T. or I.P. LPS challenges are listed in Table 2. Among these proteins, many of the same apolipoproteins (ApoA1, ApoA2, ApoA4, ApoD, ApoE), paraoxonase 1 (Pon1, involved in the regulation of lipid oxidation), S100 calcium binding protein A8 (S100A8) and tumor necrosis factor receptor superfamily member 1B (Tnfrsf1B) participate in both the LXR/RXR activation and the production of NO and ROS in macrophages. The differential regulation of these two pathways by I.T. and I.P. LPS is novel, and suggests that, while direct LPS challenge to the lung stimulates these two pathways, systemic inflammation induced by I.P. LPS suppresses lipid metabolism and inflammatory responses in lung macrophages.

The top 10 up- or down-regulated BALF proteins following I.T. or I.P. LPS administration compared to the controls are listed in Table 3 and Table 4. Interestingly, histone H2A.Z (H2afz) was the most up-regulated BALF protein following both I.T. and I.P. LPS treatment. Extracellular histones have been shown to accumulate in BALF following ALI, and their appearance requires complement 5a receptors, neutrophils and lung macrophages [18]. The sources of the extracellular histones could be dead/dying tissue cells and/or inflammatory cells, and histones are known to be associated with neutrophil extracellular traps [18]. In addition, it has been shown that, when purified histones were delivered to lung via the airways, intense inflammatory response and epithelial damage ensued [19].

### 2.3. Differential Regulation of Cxcl15 by Direct and Indirect Lung Injury

**Figure 3:**
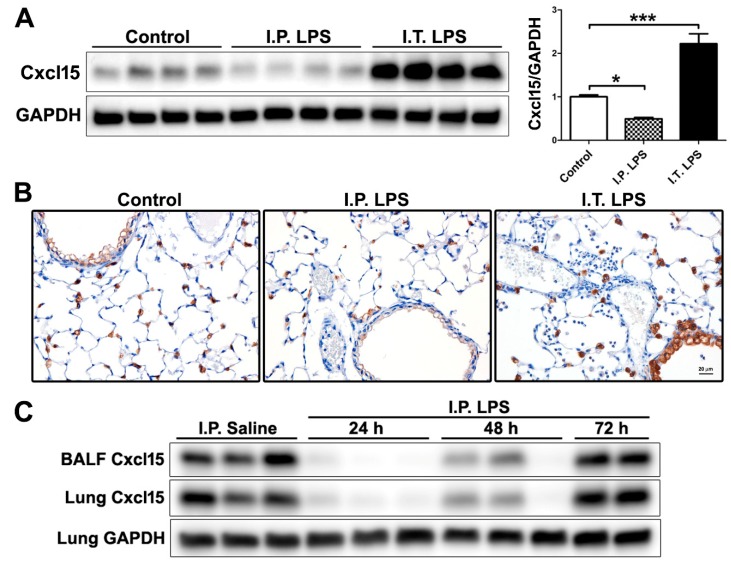
Expression of Cxcl15 in control, I.P. and I.T. LPS challenged mice. (**A**) Western blotting of lung tissue extracts. *, *p* < 0.05; ***, *p* < 0.001. Please note that protein samples from I.P. LPS group were placed next to the control group (I.T. saline) to better illustrate the down-regulation of Cxcl15 by I.P. LPS compared to the controls; (**B**) Immunostaining of Cxcl15 on lung tissue sections. Cxcl15 was expressed by both the bronchial and type II alveolar epithelial cells (brown staining). The identity of the type II alveolar epithelial cells was confirmed by immunostaining with pro-surfactant protein C, a type II cell marker, on consecutive lung tissue sections (not shown); (**C**) Expression of Cxcl15 in BALF and lung tissues following I.P. saline (48 h) and I.P. LPS treatment at different time points (24, 48 and 72 h) by Western blotting.

Among the divergently regulated proteins by I.T. and I.P. LPS (Table 1), Cxcl15 (also known as lungkine) is of particular interest. Cxcl15 was up-regulated by I.T. LPS but down-regulated by I.P. LPS, further supporting the suppression of lung innate immunity by LPS-induced systemic inflammation. Cxcl15/lungkine was first identified in lung bronchoepithelial cells and was shown to be a neutrophil chemoattractant up-regulated in various lung inflammation models [20]. The differential regulation of Cxcl15 by LPS-induced direct and indirect lung injury is intriguing. We thus performed Western blotting and immunohistochemistry to validate this proteomic finding. As shown in Figure 3A, Cxcl15 protein expression in lung tissue extracts was reduced by I.P. LPS challenge but enhanced following I.T. LPS administration, consistent with the proteomic data. Immunohistochemistry (Figure 3B) showed that Cxcl15 was constitutively expressed by both bronchial and type II alveolar epithelial cells in the control mice, and the expression of Cxcl15 was suppressed by I.P. LPS treatment but greatly enhanced by I.T. LPS. The negative controls used were sections incubated in the absence of primary antibody (monoclonal rabbit anti-mouse Cxcl15) or incubated with non-immune rabbit IgG, and both controls gave negative staining.

We further examined the down-regulation of Cxcl15 by I.P. LPS (5 mg/kg) at different time points following treatment (*n* = 3 per time point). As shown in Figure 3C, the suppression of Cxcl15 in both BALF and lung tissue protein extracts was observed as early as 24 h, and in fact, the greatest suppression was seen at this time point. The suppression of Cxcl15 was maintained at 48 h following I.P. LPS treatment. One of the three I.P. LPS treated mice died between 48 and 72 h; however, the surviving two mice seemed to be recovering at 72 h based on their improved physical appearance (posture, fur condition) and increased activity in their home cage, and with the recovering from LPS-induced systemic inflammation, Cxcl15 protein levels returned to baseline (I.P. saline control) at 72 h.

## 3. Discussion

The murine LPS model is the most commonly used preclinical model for ARDS. To our knowledge, this study is the first to examine the BALF proteome following LPS-induced direct and indirect lung injury in mice. Our quantitative discovery-based proteomic approach identified commonalities as well as significant differences in BALF protein expression profiles following LPS-induced direct and indirect lung injury. Importantly, this study identified lung-specific mechanisms of immune suppression following LPS-induced systemic inflammation. Identification and validation of these pathways in human sepsis patients are thus warranted, and strategies aimed to enhance these suppressed pathways could form effective preventive measures or treatment options for secondary lung infection in sepsis patients.

Our BALF proteomic results from I.T. LPS treated mice are in agreement with BALF proteomic analysis in human subjects challenged with I.T. LPS, as well as in human patients with pneumonia-associated ARDS. For example, ApoA1 and S100A8/9 up-regulated by I.T. LPS treatment in mice were previously found to be up-regulated in BALF in healthy human subjects challenged with LPS endotoxin (*Escherichia coli* O:113) within a lung segment and in patients with ARDS [21]. In addition, ApoA1/A2/A4 and ApoC3 up-regulated by I.T. LPS were also identified in BALF from pneumonia-associated ARDS patients in a separate study [10]. In contrast, these apolipoproteins were not increased in BALF from I.P. LPS treated mice, and in fact, ApoA4 decreased following I.P. LPS administration (Table 2). Interestingly, ApoA4 was previously identified as a down-regulated plasma protein in extra-pulmonary but not pulmonary ARDS patients [22]. In a previous proteomic study, BALF thioredoxin levels were shown to be significantly increased in ARDS non-survivors compared to survivors [11]. In our study, BALF thioredoxin level increased only in I.P. LPS group but not in the I.T. LPS group, indicating that thioredoxin might be a biomarker for survival in only a subset of ARDS patients, i.e., extra-pulmonary ARDS.

It is interesting that, although BALF protein concentration was not significantly increased in I.P. LPS treated mice, the BALF protein expression profiles of the I.P. LPS group were significantly different from those of the controls with 38 up-regulated and 85 down-regulated proteins (Supplementary Table S1). Although plasma proteins including serum amyloid proteins and hemoglobin subunit alpha were identified and found to be up-regulated in BALF from I.P. LPS treated mice, the majority of differentially regulated proteins originated from the lung including proteases/anti-proteases, as well as molecules involved in epithelial and macrophage functions. The up-regulation of plasma proteins in BALF by I.P. LPS were likely the combined results of increased vascular permeability due to LPS-induced systemic inflammatory response (including the acute phase response signaling identified by the IPA analysis) and compromised epithelial integrity as shown by down-regulation of Cadm1 and Clic5 (Table 1) and down-regulation of other molecules involved in epithelial function and cell-matrix interaction including fibulin-1, epithelial membrane protein 2 and syntenin-1 (Supplementary Table S1).

The proteomic profile of BALF from I.P. LPS treated mice revealed by the current study identified several immune pathways in the lung that were suppressed by systemic inflammation including the LXR/RXR activation pathway, the production of NO and ROS in macrophages, IL-7 signaling, and the expression of the neutrophil chemoattractant Cxcl15. The above findings were consistent with the so-called “immunoparalysis” observed in sepsis patients [23,24,25]. Severe sepsis is typically characterized by an initial cytokine-mediated hyper-inflammation or “cytokine storm”, and a subsequent hypo-inflammatory state with significant immunosuppression due to hypo-responsiveness, exhaustion, apoptotic depletion of immune cells and an increase in T regulatory and myeloid-derived suppressor cells [23,24,25]. Patients who survived early sepsis often develop nosocomial infections with organisms not typically pathogenic in immunocompetent hosts (opportunistic pathogens). A frequent site of nosocomial infection is the lung [26,27]. Our study suggests that LPS-induced systemic inflammation impairs lipid metabolism and inflammatory responses in macrophages in the lung. In addition, apolipoproteins, which participate in both LXR/RXR activation and the production of NO and ROS in macrophages, have been shown to regulate host immune responses by direct endotoxin binding and neutralization, inhibition of adhesion molecule expression, or stimulation of NO synthase production [28].

Our study also identified the IL-7 signaling pathway as one of suppressed pathways by systemic inflammation. IL-7 is a potent anti-apoptotic cytokine that enhances immune effector cell function and is essential for lymphocyte survival [29]. IL-7 has been shown to improve survival in murine models of sepsis [30], and lymphocyte functions of septic patients can be restored by ex vivo stimulation with IL-7 [31].

Cxcl15/lungkine was first identified in lung bronchoepithelial cells and was shown to be a neutrophil chemoattractant up-regulated in various lung inflammation models, including I.T. challenge with LPS and *Aspergillus* [20]. Mice lacking expression of Cxcl15 were more susceptible to *Klebsiella pneumonia* (*K. pneumonia*) infection, with decreased survival and increased lung bacterial burden compared to infected wild-type mice [32]. Histologic analysis of the lungs and assessment of leukocytes in BALF revealed that neutrophil numbers were normal in the lung parenchyma but reduced in the airspace at 24 h post *K. pneumonia* infection in Cxcl15 null mice, demonstrating that Cxcl15 is an important mediator of neutrophil migration from the lung parenchyma into the airspace [32]. The closest human gene to mouse Cxcl15 is pro-platelet basic protein (PPBP, also known as CXCL7) [20]. Increased PPBP/CXCL7 chemokine expression has been associated with neutrophil activation in patients with severe stable chronic obstructive pulmonary disease [33]. The expression and function of CXCL7 in pulmonary or extra-pulmonary ARDS, however, have not been examined.

In summary, using a quantitative discovery-based proteomic approach, this study identified commonalities as well as significant differences in BALF protein expression profiles following LPS-induced direct and indirect lung injury. Most notably, LPS-induced systemic inflammation results in suppression of select components of lung innate immunity. It remains to be determined whether the same pathways were suppressed in human sepsis patients, to what extent such changes predispose sepsis patients to subsequent infections, and whether boosting lung innate immunity would be beneficial in the management and care of sepsis patients.

## 4. Materials and Methods

### 4.1. Animals

All animal protocols were prepared in accordance with the Guide for the Care and Use of Laboratory Animals and approved by the Institutional Animal Care and Use Committee at Louisiana State University Health Sciences Center (Protocol #: 3440; Approval date: March 15, 2019). Wild-type C57BL/6 male mice at 2–3 months of age were used in the current study. Mice were fed standard chow and water *ad libitum* and maintained under 12-h day/night cycles.

### 4.2. LPS Administration

For I.T. instillation of LPS (*Escherichia coli* O111:B4; List Biological Laboratories, Campbell, CA, USA), mice (*n* = 6) were anesthetized with ketamine/xylazine (150/10 mg/kg) and intubated with a 22G catheter (EXEL International, Los Angeles, CA, USA) using the Biolite mouse intubation kit (BioTex, Houston, TX, USA). LPS reconstituted in sterile saline at 0.05 mg/mL was administered into the lungs through the 22G catheter at a final dosage of 0.1 mg/kg. For I.P. administration, LPS at 1 mg/mL in sterile saline was injected I.P. to achieve a final dosage of 5 mg/kg (*n* = 6). Mice receiving vehicle (sterile saline) alone I.T. (*n* = 3) or I.P. (*n* = 3) were used as the controls. In addition, another cohort of mice was treated with I.P. saline (*n* = 3, 48 h) or I.P. LPS (5 mg/kg, *n* = 9) and sacrificed at different time points following treatment (24, 48 and 72 h, *n* = 3 per time point).

### 4.3. Tissue Harvest, Collection and BALF Analysis

At desired time points after LPS treatment, mice were anesthetized with ketamine/xylazine and then euthanized by exsanguination through the abdominal aorta. Left lungs were tied off and snap frozen in liquid nitrogen for protein extraction. Right lungs were lavaged 3 times with 0.6 mL of phosphate-buffered saline +2 mM EDTA + protease inhibitor cocktail (Sigma, St. Louis, MO, USA). BALF was placed immediately on ice and the cellular components were separated by centrifugation at 500× *g* for 5 min at 4 °C. Supernatant from the 1st lavage was used for measurement of BALF total protein (BCA Assay; Pierce Biotechnology, Rockford, IL, USA), proteomic analysis and Western blotting. Cells from all three lavages were combined for BALF total and differential cell count (Diff-Quik; Siemens, Newark, DE, USA). Following BALF collection, right lungs were perfused with Z-Fix (Anatech, Battle Creek, MI, USA) through a tracheal cannula at a pressure of 25 cm of H_2_O for 10 min followed by fixation in Z-Fix for at least 24 h before processing for paraffin embedding and sectioning.

### 4.4. Protein Preparation and Discovery-Based Quantitative Shotgun Proteomics

Equal amount of proteins (60 μg per BALF sample) were prepared for trypsin digestion by reducing the cysteines with dithiothreitol followed by alkylation with iodoacetamide. After chloroform-methanol precipitation, each protein pellet was digested with trypsin overnight at 37 °C. The digested product was labeled using a TMT 10-plex Reagent set (Thermo Fisher Scientific, Waltham, MA) according to the manufacturer’s protocol and stored at −80 °C until further use. The 10 samples analyzed included control (I.T. saline, *n* = 3), I.T. LPS (*n* = 4) and I.P. LPS (*n* = 3).

An equal amount of each TMT-labelled sample was pooled together in a single tube and SepPak purified (Waters Chromatography, Dublin, Ireland) using acidic reverse phase conditions. After drying to completion, an off-line fractionation step was employed to reduce the complexity of the sample. The sample was brought up in 260 μL of 10 mM ammonium hydroxide, pH 10. This mixture was subjected to basic pH reverse phase chromatography (Dionex U3000, Thermo Fisher, Waltham, MA, USA). Briefly, UV monitored at 215 nm for an injection of 100 μL at 0.1 mL/min with a gradient developed from 10 mM ammonium hydroxide, pH 10 to 100% acetonitrile (ACN, pH 10) over 90 min. A total of 48 fractions (200 μL each) were collected in a 96-well microplate and recombined in a checkerboard fashion to create 12 “super fractions” (original fractions 1, 13, 25, and 37 became new super fraction #1, original fractions 2, 14, 26, and 38 became new super fraction # 2, and so on.).

The 12 “super fractions” were then run on a Dionex U3000 nano flow system coupled to a Thermo Fisher Orbitrap Fusion Tribrid Mass Spectrometer. Each fraction was subjected to a 90-min chromatographic method employing a gradient from 2–25% ACN in 0.1% formic acid (FA) over the course of 65 min, a gradient to 50% ACN/FA for an additional 10 min, a step to 90% ACN/FA for 5 min and a 10 min re-equilibration into 2% ACN/FA. Chromatography was carried out in a “trap-and-load” format using a PicoChip source (New Objective, Woburn, MA), with the trap column C18 PepMap 100 (5 μm, 100 Å) and the separation column PicoChip REPROSIL-Pur C18-AQ (3 μm, 120 Å, 105 mm). The flow rate for the entire run was at 0.3 μL/min, and electrospray was achieved at 2.6 kV.

TMT data acquisition utilized an MS3 approach for data collection. Survey scans were performed in the Orbitrap utilizing a resolution of 120,000, and data-dependent MS2 scans were performed in the linear ion trap using a collision induced dissociation of 25%. Reporter ions were fragmented using high energy collision dissociation of 65% and detected in the Orbitrap using a resolution of 50,000. This was repeated for a total of three technical replicates.

TMT data analysis was performed using Proteome Discoverer 2.2. The 3 runs of 12 “super fractions” were merged and searched using SEQUEST. The Protein FASTA database was Mus musculus (NCBIAV) version 2017-05-05. Static modifications included TMT reagents on lysine and N-terminus (+229.163), carbamidomethyl on cysteines (=57.021), and dynamic modification of oxidation of methionine (=15.9949). Parent ion tolerance was 10 ppm, fragment mass tolerance was 0.6 Da, and the maximum number of missed cleavages was set to 2. Only high-scoring peptides were considered utilizing a false discovery rate (FDR) of 1%.

The mass spectrometry proteomics data have been deposited to the ProteomeXchange Consortium (http://proteomecentral.proteomexchange.org) via the PRIDE partner repository [34,35] with the dataset identifier PXD014070 and 10.6019/PXD014070.

### 4.5. Western Blotting

Total lung proteins were extracted using RIPA buffer (Cell Signaling, Beverly, MA, USA) and quantified using the BCA Assay (Pierce Biotechnology, Waltham, MA, USA). Equal amounts of proteins from BALF or total lung protein extracts were analyzed by Western blotting as described [36]. Two Antibodies against Cxcl15 were used (goat anti-mouse Cxcl15, R & D Systems, Minneapolis, MN; monoclonal rabbit anti-mouse Cxcl15, Abcam, Cambridge, MA, USA) with similar results. Densitometry measurements were performed using NIH ImageJ.

### 4.6. Immunohistochemistry

Paraffin-embedded lung tissue sections were deparaffinized and rehydrated. Heat-induced antigen retrieval was achieved by using a pressure cooker at 98–100 °C for 15 min in 300 mM NaCl, 30 mM sodium citrate, pH 6.0, followed by cooling at room temperature for 30 min. Histostain-Plus Kits with diaminobenzidine as the substrate (Invitrogen, Carlsbad, CA, USA) was used for detection of Cxcl15 using the monoclonal rabbit anti-mouse Cxcl15 (Abcam, Cambridge, UK) per manufacturer’s instructions. Non-immune rabbit IgG (0.5 μg/mL, Invitrogen, Carlsbad, CA, USA) was used as the control. Sections were counterstained with hematoxylin.

### 4.7. Statistical Analysis and Bioinformatics

For proteomic data analysis, at least 2-fold changes (up or down) over control with an adjusted *p*-value (FDR) of less than 0.05 using the Benjamini and Hochberg procedure were considered statistically significant. IPA (Qiagen Bioinformatics, Redwood City, CA, USA) were performed to identify enriched biological processes and the most significant canonical signaling pathways.

Data were expressed as mean ±SEM. Statistical analyses were performed using GraphPad Prism. *p* < 0.05 was considered statistically significant.

## Figures and Tables

**Table 1:** Differentially regulated BALF proteins by I.T. and I.P. LPS compared to the controls.

Groups	Differentially Regulated Proteins
↑ in Both (13)	Apcs, H2afz, Hba-a1, Hist2h2aa2, Hmgn2, Itih3, Lcat, Ltf, Nucks1, Retnla, Serpina3m, Serpina3n, Tmsb4x
↓ in Both (22)	Anpep, Anxa5, AU021092, Cadm1, Cd200, Chia, Chmp5, Clic5, Cst3, Ctsc, Cyp2f2, Fam3c, Fgfr2, Il6st, Lyz1, Lyz2, Mme, Mup11, Npc2, Nrp1, Serpina7, Sftpb
↑ in I.T. and ↓ in I.P. LPS (10)	Adpgk, Apoa4, Apoe, Cxcl15, Hist1h2bp, Ighm, Igkv6-17, Lcp1, Nap1l1, Pon1
↓ in I.T. and ↑ in I.P. LPS (4)	Gsta4, Pebp1, Tppp3, Vars

BALF, bronchoalveolar lavage fluid; ↑, up-regulated; ↓, down-regulated; parenthesis, (number of proteins).

**Table 2:** Differentially regulated BALF proteins in the LXR/RXR activation and production of NO and ROS in macrophages pathways by I.T. and I.P. LPS compared to the controls.

Pathways	I.T. LPS		I.P. LPS	
	z-Score	Differentially Regulated Proteins	z-Score	Differentially Regulated Proteins
LXR/RXR Activation	1.71	Alb, ApoA1, ApoA2, ApoA4, ApoD, ApoE, C3, C4A/C4B, CD14, Clu, Fga, Gc, Il1rn, Itih4, Kng1, Lbp, Lcat, Lyz2, Mmp9, Pon1, S100A8, Tnfrsf1B	−1.07	ApoA4, ApoE, Hpx, Il1rap, Lcat, Ldlr, Lpl, Lyz1, Pltp, Pon1, Rbp4, Saa1, Serpina1, Serpinf1, Ttr
NO & ROS Production in Macrophages	1.89	Alb, ApoA1, ApoA2, ApoA4, ApoD, ApoE, Clu, Fgfr2 , Lyz2, Mapk14, Mpo, Ncf1, Ncf4, Pon1, Ppp1CA, Ppp2R1A, Ptpn6, Rhog, S100A8, Tnfrsf1B	−2.12	ApoA4, ApoE , Fgfr2, Lyz1, Mark3, Pon1, Rbp4, Serpina1

BALF, bronchoalveolar lavage fluid; LXR/RXR, liver X receptor/retinoid X receptor; NO, nitric oxide; ROS, reactive oxygen species; red, up-regulated; blue, down-regulated.

**Table 3:** Top 10 up- or down-regulated BALF proteins by I.T. LPS compared to the controls.

Up-Regulated by I.T. LPS	Down-Regulated by I.T. LPS
Histone H2A.Z (H2afz, ↑87.10)	Cell adhesion molecule 1 (Cadm1, ↓16.39)
Small nuclear ribonucleoprotein E (Snrpe, ↑55.57)	Eosinophil cationic protein 1 (Ear1, ↓15.15)
Nuclear ubiquitous casein and cyclin-dependent kinase substrate 1 (Nucks1, ↑44.40)	WAP four-disulfide core domain protein 2 (Wfdc2, ↓14.93)
Histone H3.1 (Hist1h3a, ↑37.41)	LIM zinc-binding domain-containing Nebulette (Nebl, ↓14.09)
Serine/threonine-protein kinase VRK1 (Vrk1, ↑30.05)	SEC14-like protein 2 (Sec14l2, ↓12.50)
Histone H2A type 2-A (Hist2h2aa1, ↑26.25)	Major urinary protein 18 (Mup18, ↓-10.99)
Inhibin beta A chain (Inhba, ↑23.52)	CD166 antigen (Alcam, ↓10.75)
Olfactomedin-4 (Olfm4, ↑21.75)	Lysozyme c-2 (Lyz2, ↓10.20)
Histone H3.3C (H3f3c, ↑21.04)	Cytochrome P450 2F2 (Cyp2f2, ↓9.80)
Histone H2B type 1-M (Hist1h2bm, ↑19.92)	Four and a half LIM domains protein 1 (Fhl1, ↓9.709)

BALF, bronchoalveolar lavage fluid; ↑, up-regulated; ↓, down-regulated; parenthesis, (official symbol, fold change).

**Table 4:** Top 10 up- or down-regulated BALF proteins by I.P. LPS compared to the controls.

Up-Regulated by I.P. LPS	Down-Regulated by I.P. LPS
Histone H2A.Z (H2afz, ↑15.03)	Lysozyme c-1 (Lyz1, ↓11.36)
Serum amyloid P-component (Apcs, ↑8.80)	Beta-mannosidase (Manba, ↓8.40)
Indolethylamine N-methyltransferase (Inmt, ↑8.30)	Thyroxine-binding globulin (Serpina7, ↓7.14)
Nuclear ubiquitous casein and cyclin-dependent kinase substrate 1 (Nucks1, ↑6.46)	Neuropilin-1 (Nrp1, ↓6.10)
Serum amyloid A-1 protein (Saa1, ↑6.13)	Calpain small subunit 1 (Capns1, ↓5.88)
Serine protease inhibitor A3n (Serpina3n, ↑5.62)	Ig heavy chain V region 5-84 (Ighv5-12, ↓5.75)
Resistin-like alpha (Retnla, ↑4.65)	Alpha-N-acetylgalactosaminidase (Naga, ↓5.41)
Thioredoxin-like protein 1 (Txnl1, ↑4.44)	Zinc transporter ZIP4 (Slc39a4, ↓-5.38)
Ig kappa chain V-VI region XRPC 44 (Igkv4-86, ↑4.21)	Complement C5 (C5, ↓5.38)
Acidic leucine-rich nuclear phosphoprotein 32 family member A (Anp32a, ↑4.08)	NPC intracellular cholesterol transporter 2 (Npc2, ↓5.35)

BALF, bronchoalveolar lavage fluid; ↑, up-regulated; ↓, down-regulated; parenthesis, (official symbol, fold change).

## Supplementary Materials

Supplementary materials can be found at https://www.mdpi.com/1422-0067/20/14/3401/s1. Supplementary Table S1: BALF proteome following LPS-induced direct and indirect lung injury in mice.

## Abbreviations

ACN	Acetonitrile
ALI	Acute lung injury
Apo	Apolipoprotein
ARDS	Acute respiratory distress syndrome
BALF	Bronchoalveolar lavage fluid
Cadm1	Cell adhesion molecule 1
Clic5	Chloride intracellular channel protein 5
FA	Formic acid
FDR	False discovery rate
H&E	Hematoxylin and eosin
H2afz	Histone H2A.Z
IGF	Insulin-like growth factor
I.P.	Intraperitoneal
IPA	Ingenuity Pathway Analysis
LPS	Lipopolysaccharide
I.T.	Intratracheal
Ltf	Lactotransferrin
LXR/RXR	Liver X receptor/retinoid X receptor
NO	Nitric oxide
Pon1	Paraoxonase 1
PPBP	Pro-platelet basic protein
Retnla	Resistin-like alpha
ROS	Reactive oxygen species
Sftpb	Surfactant protein B
TMT	Tandem Mass Tag
Tnfrsf1B	Tumor necrosis factor receptor superfamily member 1B
